# Toward a unified approach: Considerations for bioinformatic and sequencing activities & data in wastewater surveillance of biologic public health threats

**DOI:** 10.12688/openreseurope.20934.2

**Published:** 2026-03-10

**Authors:** Fotis Psomopoulos, Colman O’Cathail, Natasa Anastasiadou, Clementina Cocuzza, Wim L. Cuypers, Olateju Idowu, Konstantinos Kyritsis, Katharina B. Lauer, Marialuisa Lavitrano, Gabriele Leoni, Ju-Ling Liu, Guerrino Macori, Giulio Mannarà, Douglas G. Manuel, Marianna Martinelli, Rosario Musumeci, Aitana Neves, Nikos Pechlivanis, Mauro Petrillo, Jiannis Ragoussis, Nadim Rahman, Katarina Öjefors Stark, Mathew Thomson, Matthew J. Wade, Michael Wright, ELIXIR Wastewater Surveillance Working Group

**Affiliations:** 1 Institute of Applied Biosciences, Centre for Research and Technology Hellas, Thessaloniki, Greece; 2 European Molecular Biology Laboratory, European Bioinformatics Institute (EMBL-EBI), Wellcome Genome Campus, Hinxton, Cambridge, UK; 3 Department of Medicine and Surgery, University of Milano-Bicocca, Milano, Italy; 4 Adrem Data Lab, Department of Computer Science, University of Antwerp, Antwerp, Belgium; 5 University College Dublin School of Medicine, Dublin, Leinster, Ireland; 6 Airfinity Ltd, London, UK; 7 Faculty of Computer Sciences, University of Duisburg Essen, Essen, Germany; 8 Department of Medicine and Surgery, University of Milano-Bicocca, Milano, Italy; 9 European Commission, Joint Research Centre (JRC), Ispra, 21027, Italy; 10 McGill Genome Centre, Victor Phillip Dahdaleh Institute of Genomic Medicine, McGill University, Montreal, Québec, Canada; 11 Department of Human Genetics, McGill University, Montreal, Canada; 12 School of Biology and Environmental Science, University College Dublin, Dublin, Leinster, Ireland; 13 Ottawa Hospital Research Institute, Ottawa, Ontario, Canada; 14 SIB, Swiss Institute of Bioinformatics, Geneva, Switzerland; 15 Seidor Italy srl, Milano, Italy; 16 Department of Bioengineering, McGill University, Montreal, Canada; 17 Inst for Immunology, Genetics, and Pathology, SciLifeLab Data Centre, Science for Life Laboratory, Uppsala, Sweden; 18 Chief Data Officer Group, UK Health Security Agency, London, UK

**Keywords:** wastewater-based surveillance, high-throughput sequencing data, recommendations, considerations

## Abstract

Genomic technologies such as PCR and next-generation sequencing (NGS) have greatly advanced public health surveillance, especially during COVID-19, by enabling detailed tracking of pathogen spread, origins, and variants. While PCR is vital for targeted detection, falling NGS costs have made large-scale, high-throughput sequencing more feasible, supporting broader pathogen monitoring—including the detection of vaccine escape variants and new strains. Applying NGS to wastewater offers valuable population-level insights but faces challenges such as variable sample complexity, the need for skilled staff, suitable platforms, and robust IT infrastructure. Although there are currently a lot of efforts towards defining guidelines for sampling, analysis, and integrating wastewater data into public health policy, such as the recently published International Cookbook for Wastewater Practitioners, they often lack universal applicability, emphasizing the analytical approaches in favour of the NGS-based approaches. However, standardising protocols for sampling, sequencing, and analysis is crucial to ensure reliable, comparable data across surveillance systems worldwide. Pilot studies and continuous refinement are recommended to overcome implementation hurdles and fully realise the benefits of NGS in wastewater surveillance. This work attempts to outline these challenges and opportunities across the entire wastewater surveillance workflow, from data generation to reporting, and provide some concrete suggestions and considerations across the spectrum of activities.

## Plain Language Summary

Modern genomic tools like PCR and next-generation sequencing (NGS) have greatly improved how we track and study diseases, as seen during COVID-19. PCR is useful for detecting specific pathogens, while cheaper and faster NGS now allows scientists to study many pathogens at once and spot new variants, including those that might avoid vaccines.

Using NGS to analyze wastewater can give a big-picture view of community health and disease spread. However, this approach has challenges: wastewater samples can be complex, skilled staff and proper technology are needed, and strong IT systems must be in place to handle the data.

Although some guidelines exist—such as the International Cookbook for Wastewater Practitioners—they often focus more on general analysis rather than on NGS. To get reliable and comparable results worldwide, it’s important to standardize how samples are collected, sequenced, and analyzed. Pilot projects and ongoing improvements can help overcome these challenges.

This work explores these issues and opportunities throughout the whole process of wastewater surveillance and offers practical suggestions to improve each step, from collecting samples to sharing findings.

## Introduction

Genomic technologies, including polymerase chain reaction (PCR) and next-generation sequencing (NGS), have revolutionised public health surveillance, gaining significant prominence during the COVID-19 pandemic. These tools have been instrumental in illuminating pathogen dynamics, from spread and contact tracing to uncovering geographic origins and sources of infections. While PCR remains a cornerstone for targeted pathogen detection, the decreasing cost of NGS and associated computational requirements have made high-throughput sequencing increasingly accessible for large-scale surveillance programs. NGS provides a broader and more detailed understanding of pathogen genomics, enabling the detection of vaccine escape variants, recombination events, and reassortments—capabilities critical for addressing emerging public health challenges. ^1^ When applied in wastewater surveillance, NGS can offer unprecedented insights at a population scale, serving as a robust platform for monitoring both known and novel pathogens. However, establishing effective NGS and bioinformatics capability for wastewater testing presents significant challenges, including selecting appropriate sequencing platforms and methodologies, ensuring skilled personnel, and building the necessary computational and IT infrastructure. While existing guidelines offer a starting point, they are not universally adaptable, highlighting the need for tailored approaches to address the unique demands of infectious disease surveillance in diverse settings. ^1– 3^ The International Cookbook for Wastewater Practitioners – Vol. 1 SARS-CoV-2 ^4^ developed in the global response to the COVID-19 pandemic, provides a first, practical and internationally co-authored guide to wastewater-based surveillance. Developed through a unique collaboration between the European Commission, the Global Water Research Coalition, and a broad network of public and private stakeholders, it presents the rapid developments made in this field during the 2020–2023 COVID-19 pandemic health emergency. It presents best practices in sampling, analysis, and data interpretation and promotes transparent communication and integration of data from wastewater into public health policy. At the same time, it emphasises that the accuracy of NGS outcomes relies on careful attention to detail throughout the entire workflow, from sample preparation to bioinformatics analysis, and that only by optimising each step of the process, researchers can increase the confidence in their results and ensure that they are accurately representing the viral population.

Sample complexity further complicates implementation. Wastewater matrices are highly variable, containing a mix of microbial populations, environmental contaminants, and inhibitors that challenge nucleic acid extraction and amplification processes. The behaviour of pathogens and their nucleic acids in wastewater systems plays a pivotal role in shaping analytical strategies. The specific fractions of wastewater used for extraction of nucleic acids such as solids or supernatants, depending on factors such as sedimentation dynamics and pathogen morphology. Rates of decay vary across pathogens and environmental conditions, with nucleic acids degrading faster in warmer temperatures or high microbial activity zones. Shedding dynamics from human populations, influenced by factors like infection stages or individual health, add further complexity, affecting the concentration and detectability of pathogens. These pathogen-specific behaviours underscore the importance of tailoring sampling and preparation methods to optimise recovery and maximise analytical sensitivity. They are discussed further in the section “Considerations for data generators (from wet-lab to sequencing)”.

Finally, achieving standardisation across surveillance programs is vital to ensuring reproducibility and comparability of results. Developing uniform protocols for sampling, sequencing, and bioinformatics analysis would mitigate inconsistencies, particularly when scaling surveillance efforts globally. Lessons from previous technology rollouts underscore the importance of preemptive pilot studies and iterative refinement to streamline methods and avoid recurring implementation challenges. Addressing these barriers is essential to fully harness the potential of NGS for wastewater-based public health monitoring. ^3^

**Figure 1.**
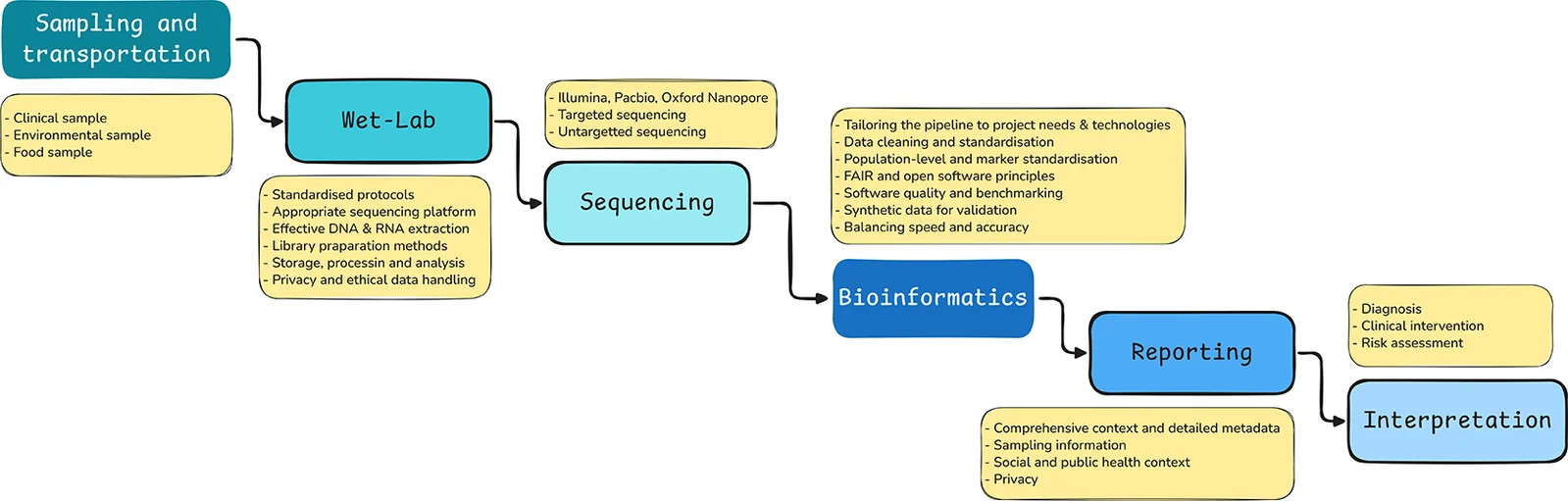
A workflow diagram that illustrates the end-to-end process (from sampling through sequencing and bioinformatics to reporting), providing a more comprehensive overview of the interconnected stages of the surveillance pipeline.

In this work we endeavour to identify the key challenges and highlight the relevant opportunities and considerations to have across the full lifecycle of wastewater data; from generation to reporting ( Figure 1), as these have been produced by the ELIXIR Wastewater Surveillance working group.

### From wet-lab to sequencing; considerations for data generators

To ensure the reliability and effectiveness of wastewater-based surveillance, several considerations must be addressed across the workflow, from sample collection to sequencing and beyond. These include careful decisions and strong metadata around: sample collection, transport and concentration protocols; the choice of sequencing technology; the methods for DNA and or RNA extraction; variability in the library preparation; the use of controls and standards; how to manage data volume; and the imperative around ethical and privacy considerations.

Standardised protocols for sample collection and processing are critical to ensuring consistency and reproducibility across studies and laboratories. Omitting details around water quality, temperature, sampling frequency or volume can make the reproducibility of results challenging. Proper handling of samples is equally important to prevent contamination and degradation, which can significantly impact downstream analyses. The time between collection and analysis may be an important factor depending on the decay kinetics of your pathogen of interest. Parameters for storage and transport, such as temperature and consistency, are also important considerations. Wastewater as a medium is highly complex and variable, and eliminating the potential for additional contaminants that may act as inhibitors is important for maintaining the integrity of the sample. Using positive and negative controls with wastewater-based analyses is encouraged, as the complex microbial and chemical profile of the base substrate can be unpredictable, and controls offer greater confidence in the validity of testing and sequencing results. Without rigorous protocols, the reliability of sequencing data may be compromised. Transparent reporting of all these parameters, where possible, will help ensure reproducibility across analyses, and more usable data.

Selecting the appropriate sequencing platform is vital, as each technology has unique strengths and limitations. Factors such as read length, accuracy, throughput, and cost must be carefully considered in the context of the study objectives and the surveillance plans. For instance, while long-read technologies may offer superior genome assembly, their cost and throughput may not always be suitable for large-scale surveillance efforts or programs in low-resource settings.

Another aspect to consider is the effective DNA and RNA extraction from wastewater samples, which can be a crucial step in the pre-analytical workflow. The efficiency of these methods directly impacts the ability to detect and characterise pathogens. Best practices for extraction should be followed to maximise yield and minimise bias, ensuring that even low-abundance pathogens are captured. It is important to support the robustness of the extraction protocols as well, including the use of both positive and negative controls or standardised mock communities to confirm the effectiveness of the methods used. The adoption of Certified Reference Materials (CRMs) like EURM-014 k ^5^ can tackle these challenges. CRMs provide a known, traceable standard that ensures the accuracy and comparability of analytical results. For example, when used as a spike, these materials can help in validating the performance of analytical methods by simulating real sample conditions, allowing laboratories to assess recovery rates, detect potential critical points in the extraction workflow, and maintain quality control. This is particularly beneficial in complex matrices like wastewater, where variability and contamination can affect data reliability. CRMs can enhance confidence in surveillance outcomes, supporting public health monitoring and environmental protection efforts.

The library preparation methods can significantly affect sequencing results and data interpretation. To address this, standard protocols or quality checkpoints should be adopted to ensure library quality. These may include qPCR designed for the amplification of adaptors included in the libraries, quantification steps, and confirming the concentration of libraries and their sizes. Negative controls should be included for the sample processing from steps of extraction, reverse transcription, or library preparation to monitor any potential contamination. Positive controls, as known pathogens introduced from steps of extraction, reverse transcription, or library preparation, can assess reagent integrity and bioinformatics pipeline performance.

Sequencing generates large volumes of data, particularly in wastewater surveillance, necessitating robust and sustainable solutions for storage, processing, and analysis. Tools and infrastructure that support efficient management and analysis of big data should be considered to streamline workflows and maximise the utility of the generated datasets.

Wastewater surveillance involves tracking pathogens in community settings, often without community members necessarily being aware. This unique aspect of wastewater surveillance thus raises concerns about privacy and ethical data handling. It is crucial to establish guidelines for ensuring that data collection and sharing respect individuals’ privacy while still providing actionable insights for public health, and we discuss this topic further in the section ‘Considerations on reporting output’.

## Considerations on the sequencing technologies

The sequencing market is currently dominated by three leading companies, namely Illumina, PacBio, and Oxford Nanopore Technologies (ONT), each providing distinct advantages catering to specific research needs. ^6^ Choosing the appropriate platform necessitates careful consideration of multiple factors, such as read quality, read length, and cost per sample, which collectively contribute to a complex decision-making process.

Illumina is widely recognised for its exceptional read quality and short-read sequencing capabilities (~100–301 bp), positioning it as the preferred platform for applications requiring high precision, such as microbial species profiling and lineage tracing. The platform’s consistent ability to produce accurate data has established it as the gold standard, particularly in studies where precision and throughput are critical. Nonetheless, Illumina’s reliance on short-read technology may render it less suitable for applications demanding longer read lengths.

In contrast, PacBio platforms excel in generating high-fidelity long reads, which are essential for complete genome assemblies and the resolution of complex genomic regions. Despite these strengths, PacBio’s limited adoption in wastewater metagenomics suggests certain drawbacks, such as higher costs.

ONT’s nanopore sequencing platforms are gaining traction due to their portability, lower upfront costs, and capability to produce long reads. In a wastewater surveillance context, these attributes make ONT platforms particularly well-suited for on-site, rapid detection tasks, such as monitoring SARS-CoV-2. Additionally, ONT’s long-read technology facilitates comprehensive microbial identification and genome assembly, especially in complex or novel pathogen scenarios. Nevertheless, the trade-off for lower initial costs and portability is often a reduction in read accuracy and data output compared to other technologies.

When it comes to sequencing strategies, two primary approaches are prevalent: targeted sequencing and untargeted sequencing (also referred to as “shotgun” metagenomics or metatranscriptomics). Targeted sequencing, often employing amplicon-based methods, is advantageous for its cost-effectiveness, as it allows for the multiplexing of numerous samples, thereby reducing overall sequencing costs. For instance, tiled-amplicon sequencing of SARS-CoV-2 from wastewater has been utilised to profile variants, enabling the analysis of a large number of samples. ^7^ However, this approach is inherently limited in scope, as it focuses only on specific targets, providing minimal to no data on other potential epidemiological targets. In addition, primer schemes need to be updated in the case of fast-evolving targets to capture adequate diversity.

On the other hand, untargeted sequencing can potentially capture the entire microbial community and/or virome present in wastewater samples, offering a broader and more comprehensive dataset. This method, however, often necessitates additional steps such as enrichment or depletion techniques to enhance the detection of low-abundant nucleic acids. For example, RNA virus identification in wastewater typically requires enrichment techniques, ^8^ while Antimicrobial Resistance (AMR) gene detection may not require biochemical enrichment. ^9^ Although untargeted sequencing yields more heterogeneous data that demands extensive bioinformatics processing, the depth of information obtained is significantly greater.

## Considerations for sequencing data

**Figure 2.**
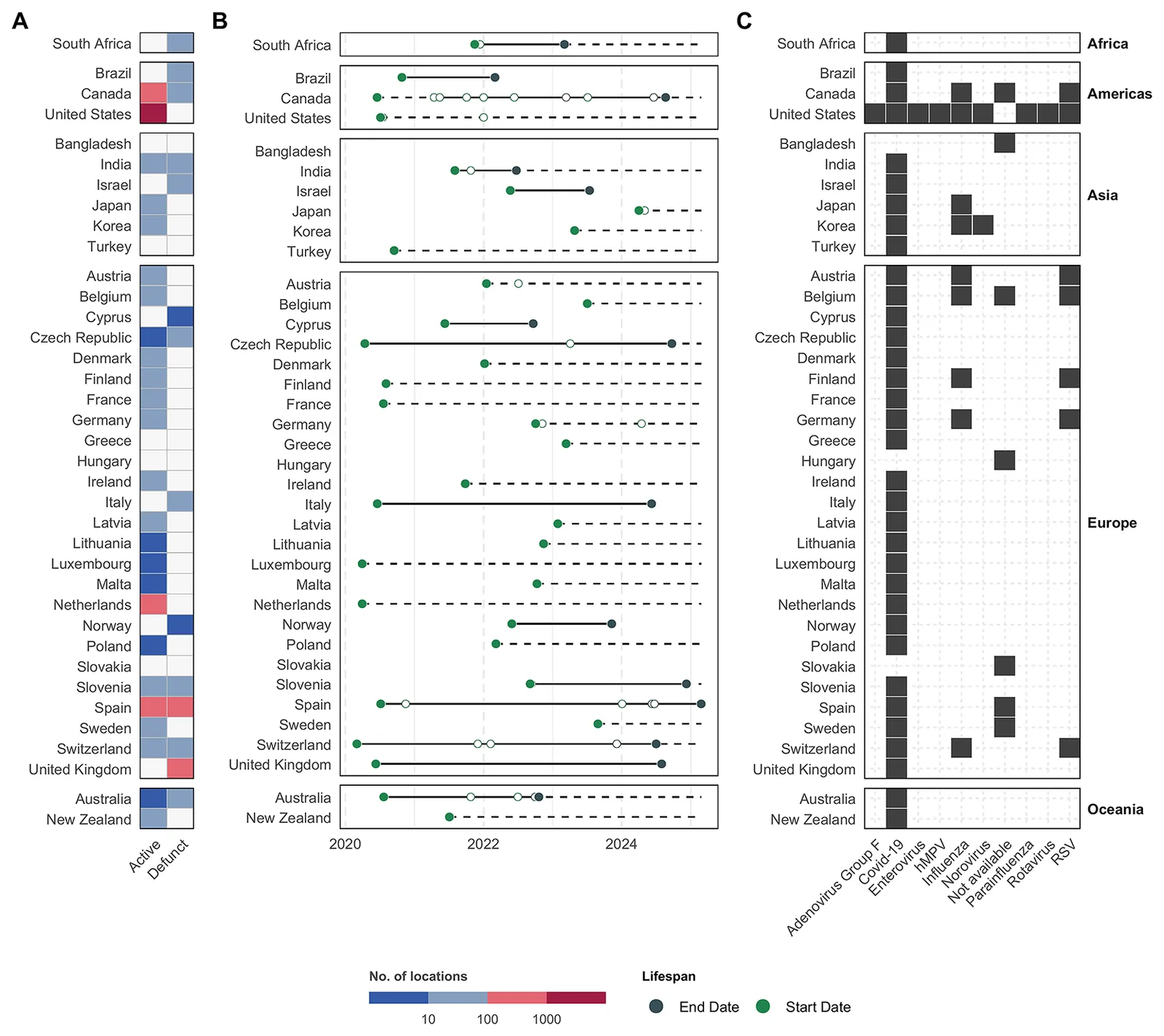
Overview of open and re-useable data wastewater surveillance initiatives (as of June 2025). **A** Active and defunct wastewater surveillance initiatives by country. **B** Duration of each initiative by country (light green indicates the start date, dark green indicates the end date, and a dashed line represents ongoing activity). **C** Diseases and viruses detectable through wastewater surveillance in each country. This non-exhaustive list highlights initiatives that make their data publicly accessible and categorises them based on current reporting status (active vs inactive). It is not intended to provide a comprehensive global overview of all wastewater surveillance efforts.

Sequencing data, irrespective of the field of investigation, has low utility without rich and well-structured metadata. Prior to starting any sequencing project, thinking about how to structure the data, what metadata you want to capture saves time downstream and helps ensure maximum utility for the future ( Figure 2). In the context of wastewater sequencing, there are several considerations of which to be aware.

The first important consideration is about sample metadata. Samples are at the core of the sequencing metadata model at the European Nucleotide Archive (ENA), and has been recommended to be central in other data object models such as the Pathogen Data Object Model (Pathogen DOM). ^10^ A “sample” in the context of a sequencing data model is a description of what was sequenced, the physical source material from which DNA or RNA was extracted for the purpose of sequencing. In wastewater, this can be a sample collected from an open drain or a sewage treatment facility.

In the International Nucleotide Sequence Database Collaboration (INSDC), there are a minimum of three metadata attributes for all samples to be registered; a taxon ID, a geographic location and/or sea, and a collection date. Sequencing investigations are often focused on sequencing the nucleotide information of a single taxon, for example, that of *Escherichia coli* or SARS-CoV-2. As a sample of wastewater is not in itself a living being, we treat it as an environment in which many living things exist, and must describe its taxonomy as such. The National Center for Biotechnology Information (NCBI) supports a rich metagenomic taxonomy tree, from which submitting users can select the most appropriate taxon ID ( https://www.ncbi.nlm.nih.gov/Taxonomy/Browser/wwwtax.cgi?id=408169).The majority of wastewater investigations will fall under taxID 527639, which represents “wastewater metagenome”.

The two other mandatory sample metadata attributes, “geographic location and/or sea” and “collection date”, are high-level descriptors to provide appropriate spatiotemporal annotation to sequencing. When describing the geographic location, users must select from a controlled vocabulary list found on the INSDC website ( https://www.insdc.org/submitting-standards/geo_loc_name-qualifier-vocabulary/).The collection date information can provide any valid date-time string that meets the ISO 8601 standard. In both cases, the INSDC recognises that there are valid reasons not to have or not be able to provide values for these attributes, and supports a missing value vocabulary for users ( https://www.insdc.org/technical-specifications/missing-value-reporting/).

Rich metadata are defined as metadata that go beyond the minimum requirements. At the ENA, a checklist system is in place to enable submitters to ensure that the metadata for their samples meets certain higher requirements. The current recommended checklist at the ENA is ERC000036 ( https://www.ebi.ac.uk/ena/browser/view/ERC000036). However, there are many standards within the community at present. The ENA endeavours to work with standards organisations such as the Genomic Standards Consortium, the Global Alliance for Genomics and Health (GA4GH), and the Public Health Alliance for Genomic Epidemiology (PHA4GE) in order to adopt community standards for sequence-related metadata.

Another major consideration for sequencing data is the structuring of samples. As described, a wastewater sample is often considered the primary sample in an investigation. Users, however, may carry out a variety of downstream work in order to create binned metagenomes, or even metagenome-assembled genomes (MAGs), from a sample. In such cases, it is recommended that users closely follow the ENA’s guidelines for structuring samples when dealing with metagenomic data submissions ( https://ena-docs.readthedocs.io/en/latest/submit/assembly/metagenome.html). This includes creating complex relationships between samples using attributes such as “sample derived from” and “sample composed of”.

Additional metadata can be crucial to report as well, though it is not mandatory in the guidelines of major repositories. For example, relevant environmental factors such as flow rate and weather conditions at the time of collection are useful to report, as these variables can significantly impact the concentration and detection of target analytes, influencing downstream analysis. Providing this information enhances the reliability of the data, supports meaningful cross-study comparisons, and is vital for improving the robustness of wastewater-based epidemiological surveillance. ^2^ Including information on the processing of wastewater samples, the choice of nucleic acid extraction protocols, and sequencing technology is also very useful for data users. Nucleic acid extraction involves isolating RNA or DNA from wastewater to detect and quantify pathogens of interest, often using magnetic bead-based systems or column-based methods to optimise yield and purity. For sequencing, high-throughput technologies such as next-generation sequencing (NGS) are typically employed to capture a broad spectrum of microbial genomes or viral RNA. Once sequenced, data analysis pipelines, including quality control, genome assembly, and variant identification, are essential for interpreting results, identifying circulating pathogens, and monitoring trends. ^3^ Transparency around what was carried out and how ensures that results are interpretable and usable to the widest possible audience.

Lastly, where possible, information on sample and data provenance should be reported. This information on who has collected and/or processed samples, or performed sequencing protocols and analysis, can contribute to others trusting the data. Particularly, if the research group, governmental agency, or other organisation who has done the work has a strong reputation. For more information on how rich metadata can best be reported alongside results, please refer to the Section on “Considerations on reporting output”.

## Bioinformatics considerations

After performing the sequencing process on the wastewater samples, and ensuring that the respective data have been assessed for quality and deposited in a FAIR repository where possible, the next step is to actually analyse the data. In this context, there are several considerations on the bioinformatics solution that one should be aware of.

### Tailoring the pipeline to project needs and technologies

First and foremost, it is crucial that the selected bioinformatics pipeline is closely tailored to meet both the specific project requirements as well as the type of sequencing or PCR technology that has been used. This is important to understand the downstream effects of certain sample processing methodologies on results which can influence sensitivity, accuracy and specificity. This involves a careful assessment of the project objectives (see also point outlined in the introduction) and the methodologies (as detailed in the section “Considerations on reporting output”). Moreover, a key requirement for the design of the bioinformatics pipeline is ensuring compatibility across the expected platform, as well as reusability.

### Data cleaning and standardisation

Another key consideration for the computational analysis is the data cleaning and standardisation; both critical steps in processing wastewater sequencing data. Given the complex and often contaminated nature of the sequencing data retrieved from wastewater samples, these steps can become significant bottlenecks. Efficiently removing contaminants, correcting errors, and standardising data formats are essential to obtaining reliable results and are pivotal in downstream analysis - while at the same time ensuring that each step is recorded in detail to enable reproducibility of the process.

### Population-level and marker standardisation

In WBE (wastewater-based epidemiology), Pepper mild mottle virus (PMMoV) and some phages (such as crAssphage or coliphages) serves as an indicator of faecal contamination and has been used to adjust pathogen concentrations, such as SARS-CoV-2, accounting for population size. However, the choice of an optimal population standardisation marker remains a subject of ongoing research, ^11, 12^ and it may be beneficial to design the pipeline to accommodate the detection of multiple markers until clear recommendations are established.

In addition to population-level standardisation, some studies have standardised the abundance of AMR markers by comparing the number of reads per marker to the estimated bacterial load, which is typically inferred from the quantification of 16S rRNA gene copies. ^13, 14^

### FAIR and open software principles

Software lies at the core of any bioinformatic activity. As such, particular care should be given towards ensuring that the software, code, and tools necessary to analyse wastewater data adhere to the community-accepted standards and recommendations.

Adhering to the FAIR (Findable, Accessible, Interoperable, and Reusable) principles is crucial when selecting bioinformatics tools. FAIR software ensures that the code and software used for the analysis is transparent and reproducible. Open workflows, which are transparent and adaptable, facilitate collaboration and innovation while ensuring that the analytical processes can be audited and replicated by other researchers. Furthermore, the low cost of using open-source software allows for a greater variety of research groups to provide wastewater data, including in situations of health emergencies. This eases the issues faced by researchers in low-resource settings who do not otherwise have the funds to use proprietary software.

Of course, FAIR doesn’t necessarily imply Open. Choosing between proprietary and open-source software involves weighing risks and opportunities. Proprietary software often offers robust support and streamlined user experiences but can be costly and less flexible. In contrast, open-source software is usually free and highly customizable, promoting transparency and community-driven improvements. It is also worth considering that while proprietary software may offer advantages, adhering to FAIR and encouraging reproducibility and open validation of results may be more difficult. There is also the issue of open-source software sometimes lacking dedicated support for users and requiring more technical expertise to implement effectively. This may be compensated for in some circumstances by the active message boards and collaborations between researchers and research groups around this software.

### Software quality and benchmarking

Beyond the FAIR and Open aspects, there are also considerations around the technical quality. High-quality research software should adhere to established quality indicators, including rigorous documentation, active maintenance, user support, and comprehensive validation against gold standard datasets. These indicators help ensure that the tools are reliable and produce credible results. There are several initiatives that focus particularly on defining both quantitative and qualitative indicators of software quality, such as the initiatives and efforts supported by EOSC, the ReSA, etc.

Another element that is critical for efficient and trustworthy bioinformatic pipelines is the performance of rigorous benchmarking. Benchmarking bioinformatics tools using reference or gold standard datasets is essential for evaluating their performance. This process helps in identifying the most accurate and efficient tools for specific tasks, such as species identification in AMR detection, ensuring that the selected tools meet the required analytical standards. Agencies like the United States National Institute of Standards and Technology (US-NIST) may help to support this work.

### Synthetic data for validation

Recently, the use of synthetic data has emerged as a versatile solution for testing and validating bioinformatics pipelines. This approach, which can involve both synthetic samples (such as the Zymo mock community), as well as simulated data (including generated sequencing reads, reads combined at specific abundances, or synthetic datasets) is designed to mimic real-world samples, allow researchers to fine-tune their methodologies and assess the performance of their tools in a controlled environment without the limitations and variability of actual wastewater samples.

Various databases and tools are available for identifying species and detecting AMR in sequencing data. For instance, AMRFinder and ResFinder are specialised tools for AMR gene detection. These databases need to be current, comprehensive, and well-curated to provide accurate and actionable insights.

### Balancing speed and accuracy

Finally, part of the benchmarking activity is an assessment of the trade-off between speed and accuracy in bioinformatics analyses. While faster tools can provide quicker results, they may sacrifice accuracy. Conversely, highly accurate tools might be slower and computationally intensive. Selecting the appropriate tools involves balancing these factors based on the specific needs and constraints of the project.

As an example, activities such as metagenome assembly, k-mer matching, and read mapping are fundamental techniques in processing sequencing data from wastewater. These methods must be optimised and validated to ensure they can handle the complexity and diversity of wastewater samples. Benchmarking these techniques against established datasets helps verify their effectiveness and reliability.

## Considerations on reporting output

When reporting wastewater sequencing data, it is crucial to provide comprehensive context and detailed metadata to enable meaningful analysis and ensure the data can be effectively reused. In light of the ongoing reproducibility crisis in science, where studies often cannot be replicated due to insufficient information about how data was generated, transparency in reporting is essential. Wastewater sequencing, although gaining significant traction during the COVID-19 pandemic, is still a relatively new tool for large-scale surveillance. For this data type to evolve into a routine surveillance method, it is vital to report all necessary details to allow others to replicate findings, innovate, and build upon the work. Ensuring thorough documentation of methods and metadata will ultimately strengthen the scientific value and long-term utility of wastewater surveillance data ( Figure 2).

When reporting wastewater sequencing data, it is essential to include detailed sampling information alongside the results to ensure accurate interpretation and comparability. Key details, as discussed in the section “Considerations for sequencing data”, should include the location, date, and time of sampling (given that transportation conditions of sampling and testing at central vs satellite laboratories can also influence reliability). Reporting environmental and protocol information about the sample and the analyses also maximise the usefulness of the data being reported ( Table 1).

**Table 1.** Checklist of the suggested requirements for a report.

#	Category	Description
1	Sampling information	Location
2		Date
3		Time of sampling
4		Environmental metadata
5		Extraction method
6		Controls
7	Social and public health context	Population size
8		Outbreaks
9		Public health data

When reporting the results, knowing and sharing the social and public health context is crucial: reporting the population size served by the wastewater system, any known outbreaks, and other local public health data helps contextualise the findings. Data standardisation, such as adjusting for variations in sample concentration or flow rate, is also key to ensuring that trends in pathogen levels are reflective of true epidemiological dynamics rather than fluctuations in sampling conditions. ^15^ These methodological and environmental details must be meticulously documented to ensure reproducibility and meaningful comparison across studies. Further, when reporting results and outputs from wastewater analysis, it is essential that they are visualised and presented in formats that are easy to reuse and follow widely adopted standards. This ensures consistency and interoperability across studies, and also facilitates machine-readable formats for automated data processing and analysis. Standard-setting consortia and organisations should be considered when designing these outputs, including the Genomic Standards Consortium (GSC), the United States Centres for Disease Control (US-CDC)‘s National Wastewater Surveillance System (NWSS), the Public Health and Environmental Surveillance Open Data Model (PHES-ODM), the Global Water Pathogen Project, the Wastewater-Based Epidemiology (WBE) Collaborative, or the Sewage Analysis CORe group Europe (SCORE).

Wastewater-based testing presents a unique opportunity for public health monitoring by capturing data from symptomatic, asymptomatic, and pre-symptomatic individuals without relying on clinical testing, but it also raises significant ethical concerns. One of the key issues is privacy, as individuals may not be aware they are being monitored, raising questions about informed consent. Transparency, documentation, open reporting and clear communication about the use and benefits are essential to maintain public trust and protect the anonymity of any individuals who may be impacted by WBE reporting. Additionally, approaches to ensure that data are as open as possible and as closed as necessary should be taken when reporting results to prevent misuse, especially as technology advances to potentially include human-specific biomolecules. ^16, 17^ One further emerging concern is around the possibility of identifying individuals from WBE data. The rapid advancements being made in the field are making WBE data more useful and applicable, and enriching what information can be gathered from this surveillance modality. The corollary to this advancement is that the possibility of linking WBE data back to individuals becomes increasingly possible. ^18^ While many research ethics boards refuse to consider WBE studies because there is no re-identification of human subjects or information collected on individuals, picking up fragments of human DNA when sequencing from wastewater is a reality. The USCDC has already put policies in place around using de-hosting/human read removal tools, and also not publicly reporting data on dashboards for catchment areas smaller than 3,000 people. ^19^ While WBE researchers have claimed the population sizes under study have added an extra layer of anonymity to surveillance results, but in smaller sewersheds the natural variation in shedding dynamics of individuals may introduce greater variation and compromise data quality. ^20^ More importantly from an ethical perspective this variation between individuals and across pathogens means that a population size cutoff for publicly reporting data may vary across pathogens and sewershed types, and may need to be revisited as WBE technology continues to advance. Ethical reviews should ensure that wastewater-based testing balances public health benefits with individual rights, aligning with legal frameworks and preventing scientific misuse while not closing the door on innovation in this promising field of surveillance. ^21, 22^ It will also continue to be imperative that best practices in genomic data privacy continue to be applied to WBE data.

## Conclusions

The future of wastewater NGS surveillance lies in its seamless integration into routine public health monitoring systems, including syndromic surveillance networks, enabling near real-time detection of pathogens and early warning of outbreaks. Achieving this vision necessitates the standardisation of methods across sampling, sequencing, and bioinformatics workflows. Learning from the challenges of previous technologies, accelerated standardisation can be realised through fostering global collaboration, developing adaptable protocols, and leveraging open-access data platforms. Addressing recurring “teething issues” when new technologies emerge requires pre-emptive pilot studies and iterative feedback loops to refine methodologies. Streamlined, standardised approaches will enhance the scalability, comparability, and impact of wastewater NGS in safeguarding public health. Recent frameworks such as PathoSeq-QC ^23^ underscore the importance of standardising the entire sequencing workflow—from sample collection to bioinformatics analysis. They promote pathogen enrichment, the use of both short- and long-read sequencing, and the development of dedicated tools and curated databases for accurate monitoring. These comprehensive approaches can enhance the scalability, sensitivity, and reliability of WBE, and contribute to position it as a vital tool for real-time public health surveillance and early outbreak detection.

A key driver for advancing wastewater surveillance adoption is its cost-effective, real-time insight into population health, which plays a crucial role in building more inclusive public health systems, especially at a time when public healthcare budgets face constant cuts. The launch of the Global Consortium for Wastewater and Environmental Surveillance for Public Health (GLOWACON) by the European Commission’s Health Emergency Preparedness and Response Authority (HERA), in collaboration with the Joint Research Centre (JRC), marks a pivotal step toward institutionalising wastewater surveillance globally ( https://health.ec.europa.eu/latest-updates/launching-glowacon-global-initiative-wastewater-surveillance-public-health-2024-03-21_en). GLOWACON aims to establish an international sentinel system for early detection and real-time monitoring of epidemic threats by integrating wastewater data with community-based surveillance, particularly at strategic locations like airports. With participation from over 300 global stakeholders—including WHO, the Africa CDC, and the U.S. CDC—the initiative fosters cross-sectoral collaboration, capacity building, and innovation. By aligning technical protocols, data integration strategies, and funding mechanisms, GLOWACON is expected to significantly enhance global health security and pandemic preparedness.

Through coordinated global initiatives like GLOWACON aimed to standardise methodologies, and promote open, collaborative innovation, wastewater-based surveillance can become a cornerstone of resilient, equitable, and proactive public health systems worldwide.

### Ethics and consent

Ethical approval and consent were not required.

## Data Availability

All data used for the creation of Figure 1 are available on Zenodo ^24^: https://doi.org/10.5281/ZENODO.15876932 Data are available under the terms of the Creative Commons Attribution 4.0 International.
