# Supplementary material for: Toward a unified approach: Considerations for bioinformatic and sequencing activities & data in wastewater surveillance of biologic public health threats
Source: Open Res Eur. 2026 Jul 6;5:267. Originally published 2025 Sep 2. [Version 3] doi: 10.12688/openreseurope.20934.3 (PMC13550913; doi:10.12688/openreseurope.20934.3)
Supplement: Peer review history

# Reviewer report: Reviewer response for version 3

**DOI:** 10.21956/openreseurope.26469.r76962

**Published:** 2026-07-09

**Authors:** Russell Faust

**Affiliations:** 1 University of Illinois Chicago, Chicago, Illinois, USA

Disappointingly, many of my original concerns remain unaltered: 

 "The manuscript would benefit from revision to align with conventional scientific vernacular. Several instances of informal phrasing should be corrected—for example, 'a lot of efforts' → 'numerous efforts'; 'the NGS-based ones' → 'NGS-based protocols/approaches'; 'big-picture view' → 'population-level perspective.' Additionally, the phrase describing variants that 'might avoid vaccines' anthropomorphizes viral behavior; the established terminology is 'vaccine escape variants' or 'variants capable of immune evasion.'" This language is unchanged and still inappropriate in a scientific manuscript.

 The ethics section "doesn't engage with the emerging literature on granularity thresholds—at what population size does wastewater surveillance become potentially re-identifiable? This is an ongoing concern for every public health jurisdiction that employs wastewater surveillance in the US." This remains unaddressed.

Where applicable, are recommendations and next steps explained clearly for others to follow? (Please consider whether others in the research community would be able to implement guidelines or recommendations and/or constructively engage in the debate)

Partly

Does the article adequately reference differing views and opinions?

Yes

Are all factual statements correct, and are statements and arguments made adequately supported by citations?

Yes

Is the rationale for the Open Letter provided in sufficient detail? (Please consider whether existing challenges in the field are outlined clearly and whether the purpose of the letter is explained)

Yes

Is the Open Letter written in accessible language? (Please consider whether all subject-specific terms, concepts and abbreviations are explained)

Partly

Reviewer Expertise:

Wastewater-based epidemiology; Public Health Informatics; DNA Sequencing Methods; Genomic Epidemiology

I confirm that I have read this submission and believe that I have an appropriate level of expertise to confirm that it is of an acceptable scientific standard, however I have significant reservations, as outlined above.

# Reviewer report: Reviewer response for version 2

**DOI:** 10.21956/openreseurope.25213.r70997

**Published:** 2026-03-31

**Authors:** Aditi Nag

**Affiliations:** 1 Dr. B. Lal Institute of Biotechnology, Jaipur, Rajasthan, India

This work has attempted to outline challenges and opportunities across the entire wastewater surveillance workflow, from data generation to reporting, and provide some concrete suggestions and considerations across the spectrum of activities. 

 The letter has apt description of the gaps in wastewater surveillance and challenges thereof. It is a comprehensive attempt to identify and discuss the various steps and variables which affect the surveillance. The process variations of sample collection to pre-processing to molecular analysis to bioinformatics assessments have also been described. The question raised on uniformity of tests and reporting is a valid and worth exploring question. However, in this manuscript it seems that a definitive and conclusive unified guideline or protocol is starkly missing. In absence of such a protocol, this manuscript is just another assessment added to the previous literature which asks for a standard guideline. One major discussion which I found inadequately discussed was how the infrastructure and government policies (funds and other policies) affect this surveillance.

 This can however serve as a good introduction manuscript for the domain of wastewater based epidemiology.

Where applicable, are recommendations and next steps explained clearly for others to follow? (Please consider whether others in the research community would be able to implement guidelines or recommendations and/or constructively engage in the debate)

Not applicable

Does the article adequately reference differing views and opinions?

Partly

Are all factual statements correct, and are statements and arguments made adequately supported by citations?

Yes

Is the rationale for the Open Letter provided in sufficient detail? (Please consider whether existing challenges in the field are outlined clearly and whether the purpose of the letter is explained)

Yes

Is the Open Letter written in accessible language? (Please consider whether all subject-specific terms, concepts and abbreviations are explained)

Yes

Reviewer Expertise:

Wastewater based epidemiology, AMR and COVID-19 surveillance in wastewater.

I confirm that I have read this submission and believe that I have an appropriate level of expertise to confirm that it is of an acceptable scientific standard, however I have significant reservations, as outlined above.

# Reviewer report: Reviewer response for version 2

**DOI:** 10.21956/openreseurope.25213.r70870

**Published:** 2026-03-27

**Authors:** Sandra Martinez-Puchol

**Affiliations:** 1 Universitat de Barcelona, Barcelona, Spain

I have now reviewed the revised version of the manuscript, and I am satisfied with the changes made by the authors. I believe that the revisions adequately address the concerns previously raised, and the addition of the table, figure, and clarifying text significantly improves the manuscript.

 In my opinion, the article is now suitable for indexing, and I am happy to update my previous status accordingly.

Where applicable, are recommendations and next steps explained clearly for others to follow? (Please consider whether others in the research community would be able to implement guidelines or recommendations and/or constructively engage in the debate)

Partly

Does the article adequately reference differing views and opinions?

Yes

Are all factual statements correct, and are statements and arguments made adequately supported by citations?

Yes

Is the rationale for the Open Letter provided in sufficient detail? (Please consider whether existing challenges in the field are outlined clearly and whether the purpose of the letter is explained)

Partly

Is the Open Letter written in accessible language? (Please consider whether all subject-specific terms, concepts and abbreviations are explained)

Yes

Reviewer Expertise:

Viral NGS, WBE

I confirm that I have read this submission and believe that I have an appropriate level of expertise to confirm that it is of an acceptable scientific standard.

# Reviewer report: Reviewer response for version 1

**DOI:** 10.21956/openreseurope.22648.r64311

**Published:** 2025-11-25

**Authors:** Russell Faust

**Affiliations:** 1 University of Illinois Chicago, Chicago, Illinois, USA

The manuscript “Toward a unified approach: Considerations for …” does address a needed topic.

 The rationale for the Open Letter is clearly stated.

 The article does adequately reference differing views.

 Factual statements are correct and adequately supported.

 “Next steps” are overly vague: “pilot studies are recommended” and “standardization is important” adds little value to the literature.

 No competing interests were disclosed.

**Reviewer Expertise**: Wastewater-based epidemiology; Public Health Informatics; DNA Sequencing Methods; Genomic Epidemiology

 I confirm that I have read this submission and that I have an appropriate level of expertise to assess scientific content of the manuscript.

 I have significant reservations regarding the value of the manuscript for publication, as noted here. Aside from the minimal value added, the authors may wish to engage a scientific editor familiar with infectious disease nomenclature, as noted in further detail below.

**Article Strengths**:

 The piece attempts something genuinely needed—addressing the standardization gap for NGS in wastewater surveillance that the International Cookbook largely sidesteps. The workflow-based organization (wet lab → sequencing → bioinformatics → reporting) is pedagogically sound and mirrors how you structured your own curriculum work. The metadata discussion citing ENA checklists, INSDC standards, and PHA4GE is appropriately granular, and the acknowledgment of FAIR principles for bioinformatics software reflects current best practices.

 Figure 1 provides a useful snapshot of global surveillance initiatives, and the discussion of GLOWACON positions wastewater surveillance within the broader pandemic preparedness architecture—relevant given HERA's involvement.

**Article Weaknesses**:

 The fundamental question is: what is the contribution here? It's labeled an "open letter," which occupies an ambiguous space between commentary and guidance document. The recommendations remain frustratingly general—"standardization is important," "pilot studies are recommended"—without the prescriptive specificity that would make this operationally useful.

**Other gaps**: there is no discussion of integration with clinical genomic surveillance, limited treatment of cost-effectiveness thresholds for NGS versus targeted PCR in resource-constrained settings, and the sequencing platform comparison (Illumina/PacBio/ONT) could go deeper on wastewater-specific performance data rather than general platform characteristics.

 The ethics section, while appropriately included, doesn't engage with the emerging literature on granularity thresholds—at what population size does wastewater surveillance become potentially re-identifiable? This is an ongoing concern for every public health jurisdiction that employs wastewater surveillance in the US.

**Finally**:

 The manuscript would benefit from revision to align with conventional scientific vernacular. Several instances of informal phrasing should be corrected—for example, 'a lot of efforts' → 'numerous efforts'; 'the NGS-based ones' → 'NGS-based protocols/approaches'; 'big-picture view' → 'population-level perspective.'

 Additionally, the phrase describing variants that 'might avoid vaccines' anthropomorphizes viral behavior; the established terminology is 'vaccine escape variants' or 'variants capable of immune evasion.' The authors may wish to engage a scientific editor familiar with infectious disease nomenclature.

Where applicable, are recommendations and next steps explained clearly for others to follow? (Please consider whether others in the research community would be able to implement guidelines or recommendations and/or constructively engage in the debate)

Partly

Does the article adequately reference differing views and opinions?

Yes

Are all factual statements correct, and are statements and arguments made adequately supported by citations?

Yes

Is the rationale for the Open Letter provided in sufficient detail? (Please consider whether existing challenges in the field are outlined clearly and whether the purpose of the letter is explained)

Yes

Is the Open Letter written in accessible language? (Please consider whether all subject-specific terms, concepts and abbreviations are explained)

Partly

Reviewer Expertise:

Wastewater-based epidemiology; Public Health Informatics; DNA Sequencing Methods; Genomic Epidemiology

I confirm that I have read this submission and believe that I have an appropriate level of expertise to state that I do not consider it to be of an acceptable scientific standard, for reasons outlined above.

# Reviewer report: Reviewer response for version 1

**DOI:** 10.21956/openreseurope.22648.r59838

**Published:** 2025-10-08

**Authors:** Sandra Martinez-Puchol

**Affiliations:** 1 Universitat de Barcelona, Barcelona, Spain

The manuscript *“Toward a unified approach: Considerations for bioinformatic and sequencing activities & data in wastewater surveillance of biologic public health threats”* addresses a highly relevant and timely topic. Wastewater-based epidemiology (WBE) using next-generation sequencing (NGS) is an emerging cornerstone of global public health surveillance. The article is written as an Open Letter, aiming to provide a broad overview of considerations for sequencing and bioinformatics in WBE.

 Overall, the paper presents a comprehensive outline of the challenges and opportunities in this field. However, some areas could be strengthened to enhance clarity, utility for practitioners, and long-term impact. I recommend minor revisions before indexing.

- Including a workflow diagram illustrating the end-to-end process (from sampling through sequencing and bioinformatics to reporting) would provide a more comprehensive overview and help readers visualise the interconnected stages of the surveillance pipeline.
- From wet-lab to sequencing (p. 4) - Recommendations are generic in places. E.g in “Proper handling of samples is equally important to prevent contamination and degradation…” it would be beneficial to expand this point by discussing the presence of inhibitors and complex microbial mixtures, the adoption of negative controls at each step, and the importance of transparent reporting.
- Reporting output (pp. 8–9) - Provide a checklist in this section to address all the suggested requirements for a report (Sampling site, date, and time, Environmental metadata, Extraction method, controls...).

Where applicable, are recommendations and next steps explained clearly for others to follow? (Please consider whether others in the research community would be able to implement guidelines or recommendations and/or constructively engage in the debate)

Partly

Does the article adequately reference differing views and opinions?

Yes

Are all factual statements correct, and are statements and arguments made adequately supported by citations?

Yes

Is the rationale for the Open Letter provided in sufficient detail? (Please consider whether existing challenges in the field are outlined clearly and whether the purpose of the letter is explained)

Partly

Is the Open Letter written in accessible language? (Please consider whether all subject-specific terms, concepts and abbreviations are explained)

Yes

Reviewer Expertise:

Viral NGS, WBE

I confirm that I have read this submission and believe that I have an appropriate level of expertise to confirm that it is of an acceptable scientific standard, however I have significant reservations, as outlined above.
